# Supplementary material for: Genome-wide CRISPR screens of oral squamous cell carcinoma reveal fitness genes in the Hippo pathway
Source: eLife. 2020 Sep 29;9:e57761. doi: 10.7554/eLife.57761 (PMC7591259; doi:10.7554/eLife.57761)
Supplement: Supplementary file 8. [file elife-57761-supp8.pdf]

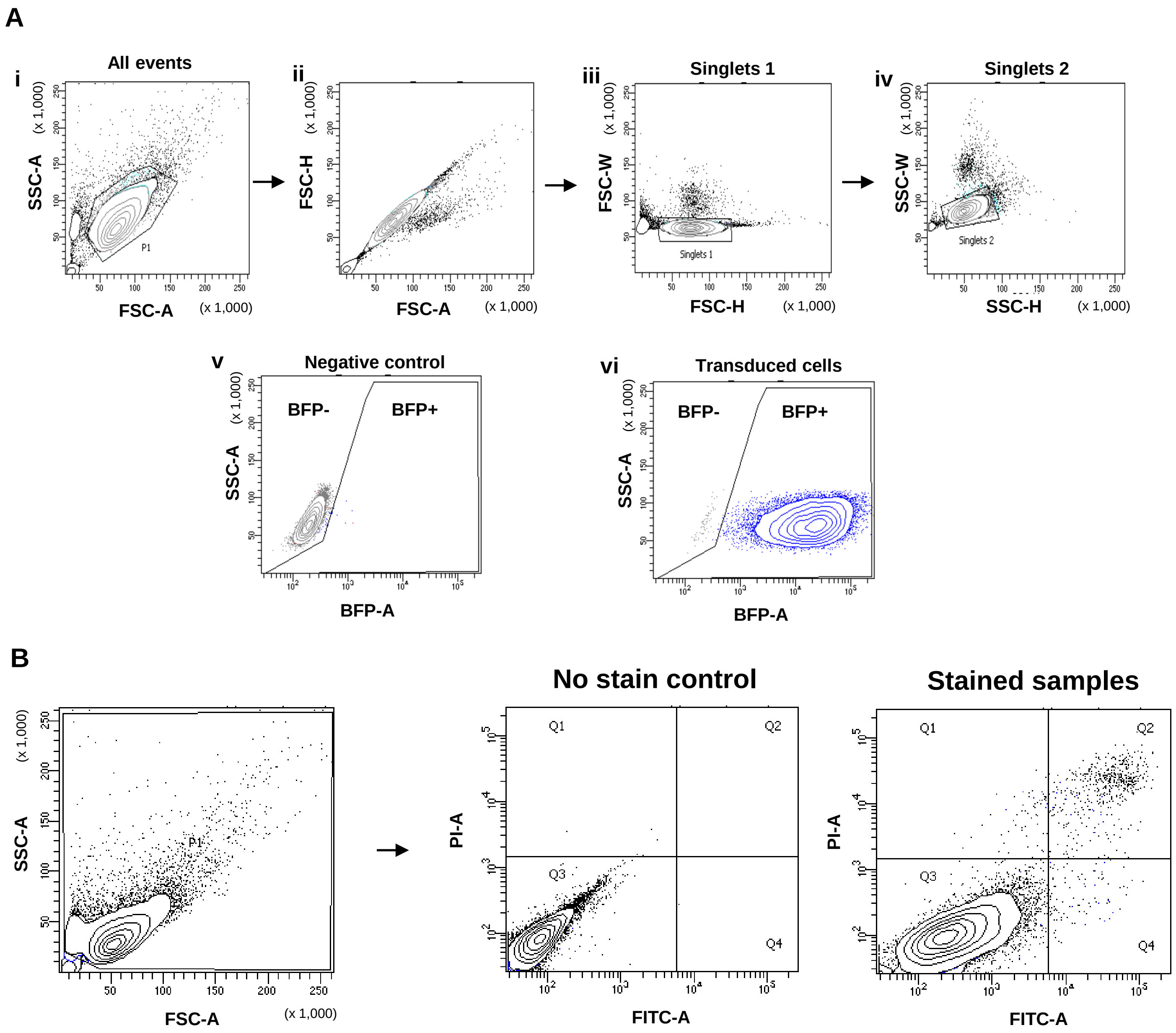

Supplementary file 8 Representative figures exemplifying gating strategies for detection of fluorescence marker-expressing cells and apoptotic cells using flow cytometry analysis. **(A)** Cells were first gated to exclude debris (i), area scaling is adjusted such that main population (P1) FSC-H and FSC-A are proportional (ii), further gatings were applied to select for singlets (iii, iv). Percentage of BFP+ or other fluorescence marker-expressing cells will be estimated using negative control (v) to guide the gate position (vi). **(B)** Cells were first gated to exclude debris (P1), then plot of PI-A and FITC-A was used to determined percentage of single-stained (Q1/Q4) and double-stained cells (Q2) – total apoptotic cells (including early apoptotic, late apoptotic and necrotic cells). No stain control was included to guide gating, all unstained or non-apoptotic cells are found in Q3.
