# Supplementary material for: Genome-wide CRISPR screens of oral squamous cell carcinoma reveal fitness genes in the Hippo pathway
Source: eLife. 2020 Sep 29;9:e57761. doi: 10.7554/eLife.57761 (PMC7591259; doi:10.7554/eLife.57761)
Supplement: Supplementary file 13. [file elife-57761-supp13.pdf]

Supplementary file 13 – All uncropped western blot images.

For Figure 3B: Single Knockout (1)

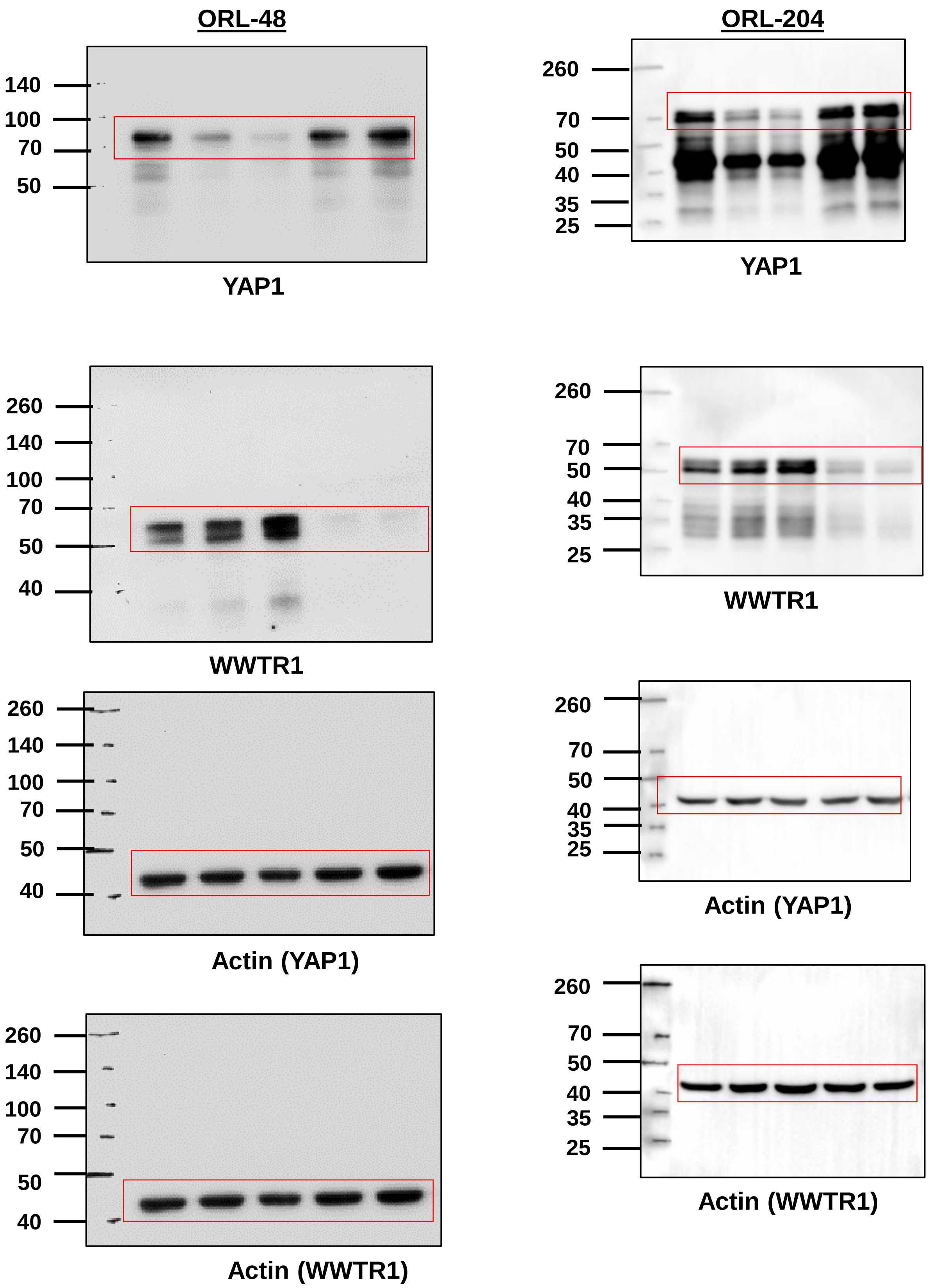

For Figure 3B: Single Knockout (2)

ORL-214

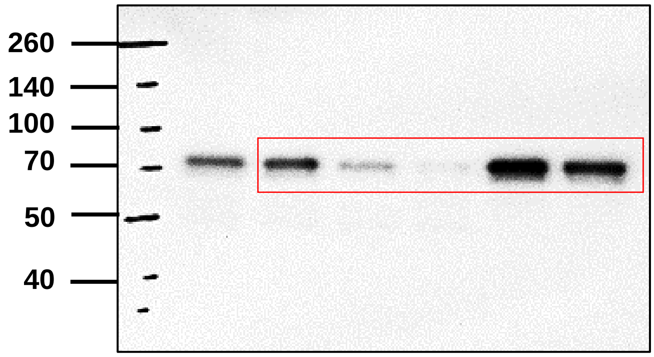

YAP1

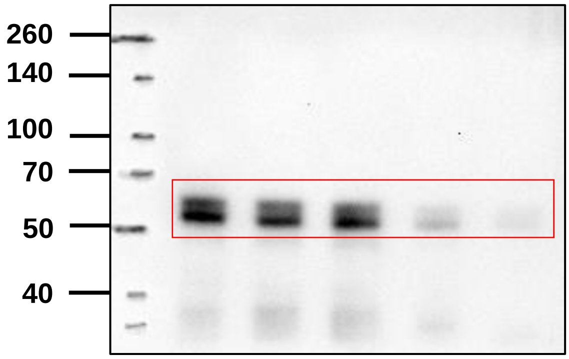

WWTR1

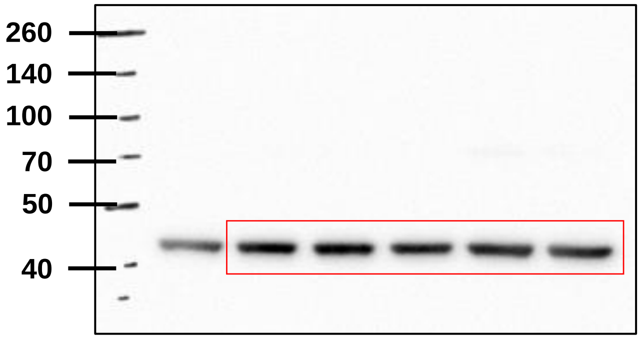

Actin (YAP1)

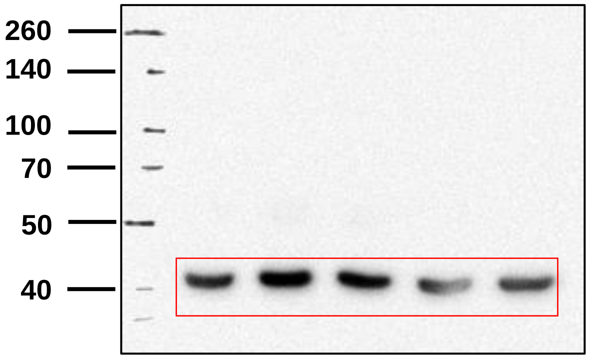

Actin (WWTR1)

PE/CA-PJ15

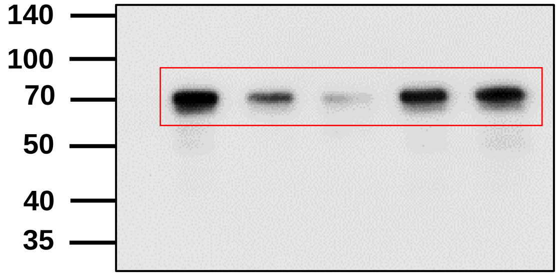

YAP1

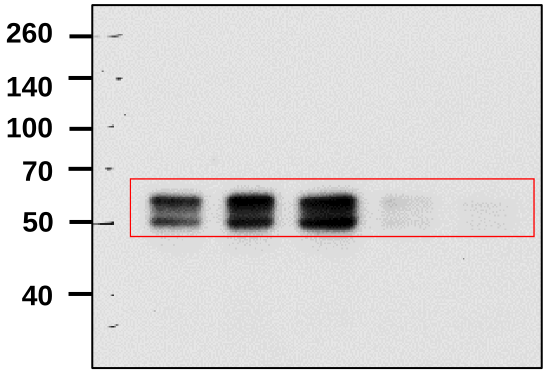

WWTR1

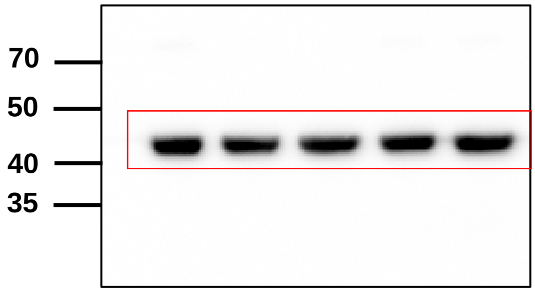

Actin (YAP1)

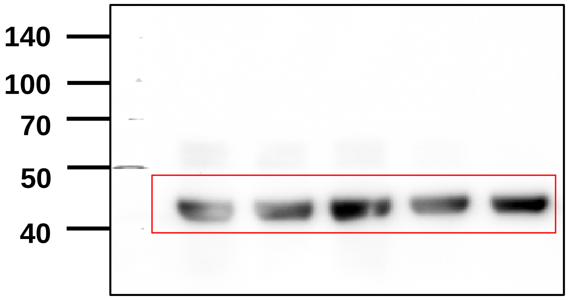

Actin (WWTR1)

For Figure 3B: Single Knockout (3)

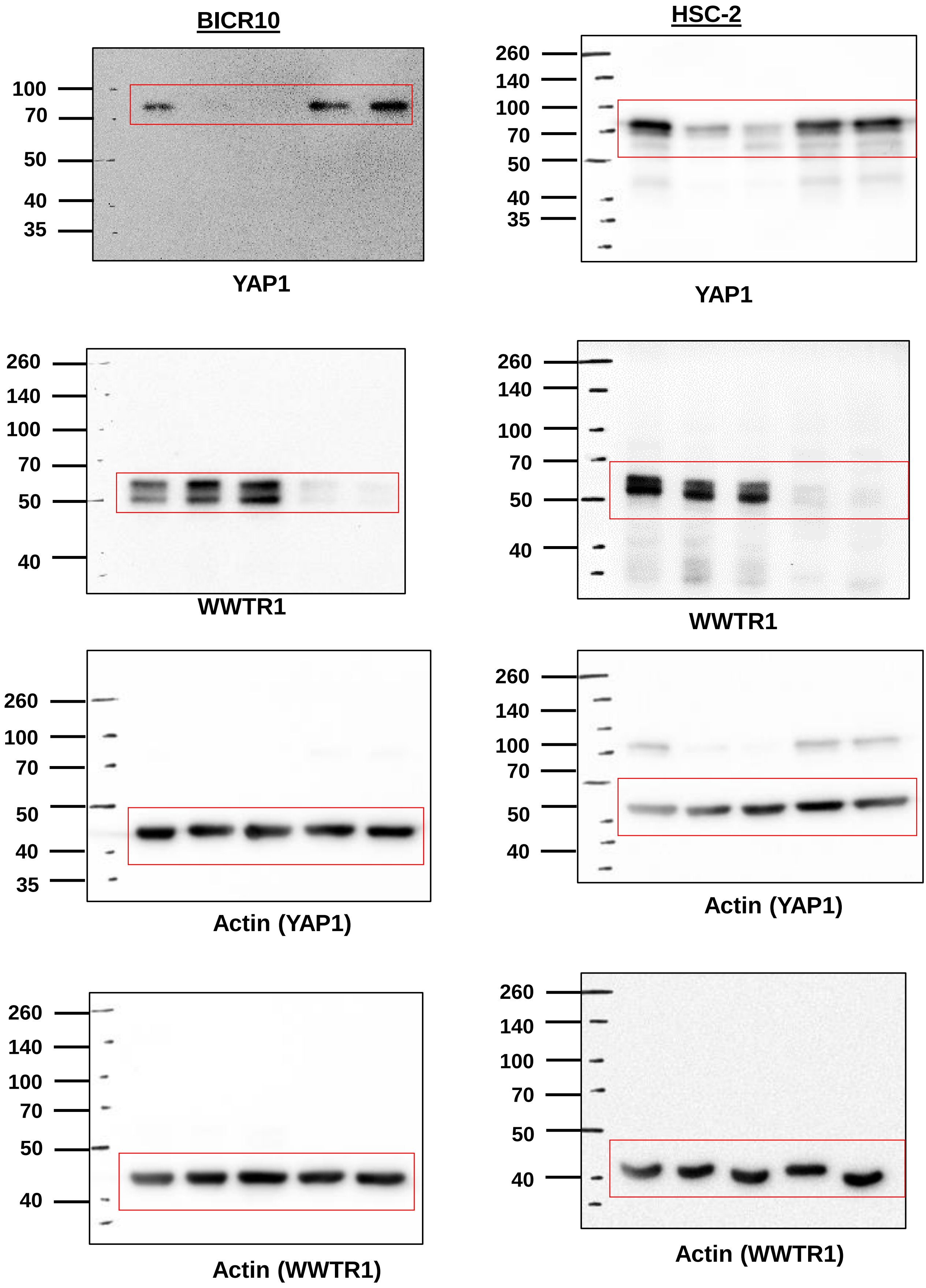

For Figure 3-figure supplement 2F: Parental Baseline

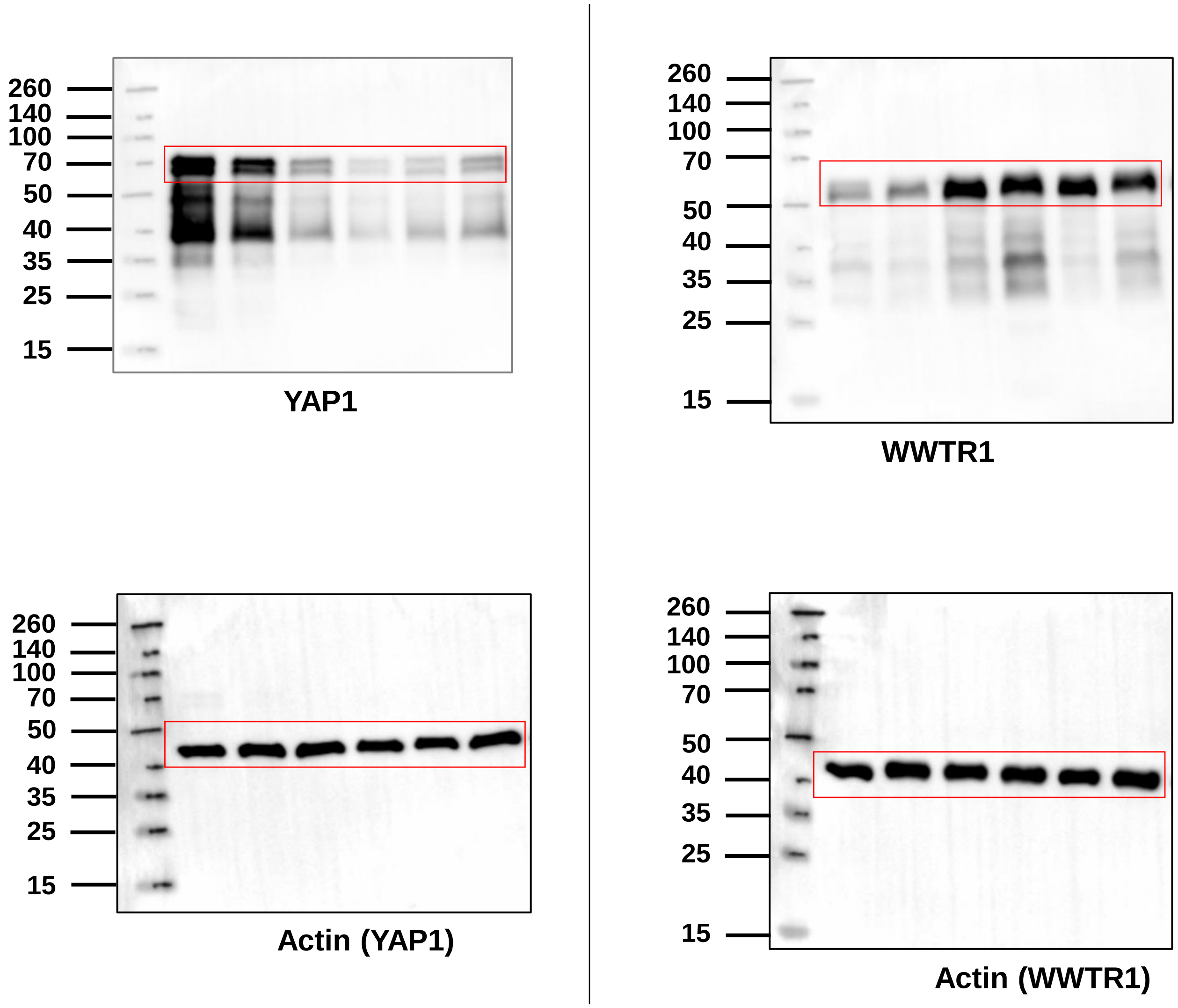

For Figure 3-figure supplement 4C: Single Knockout

SAS

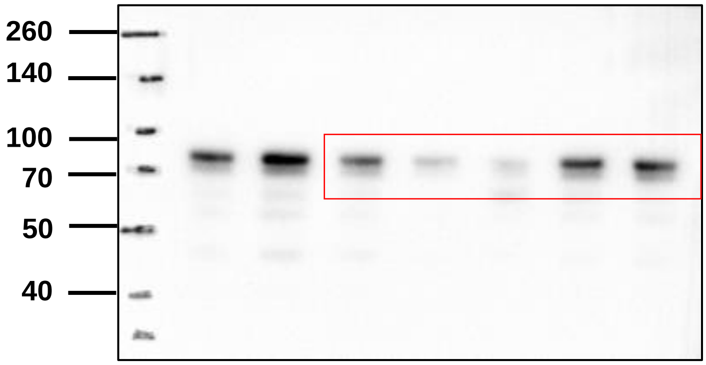

YAP1

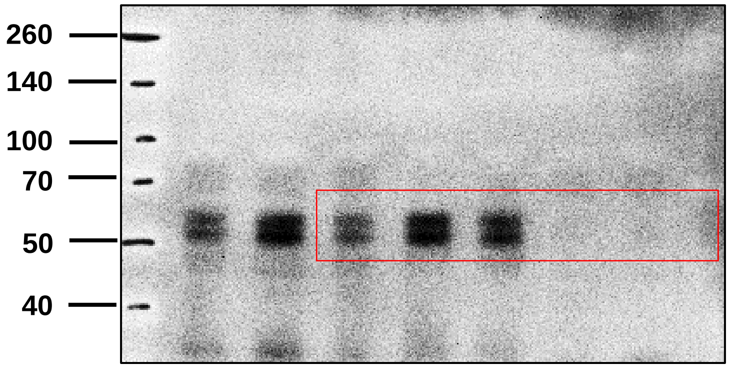

WWTR1

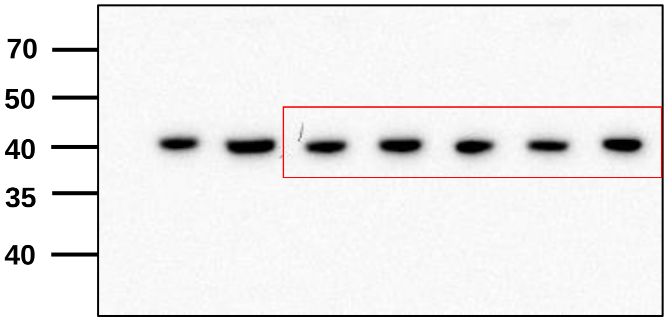

Actin (YAP1)

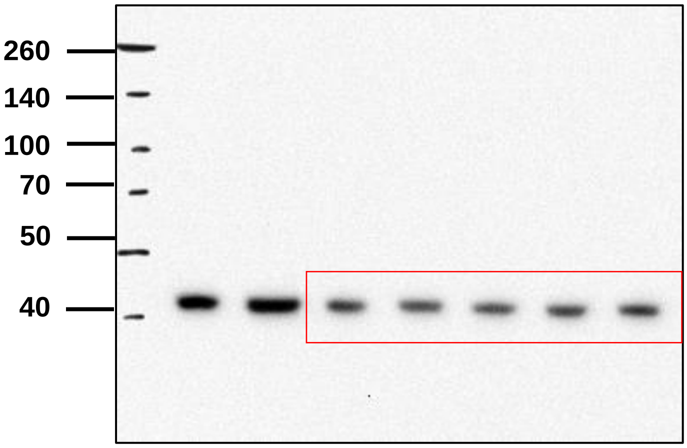

Actin (WWTR1)

For Figure 5: Double Knockout

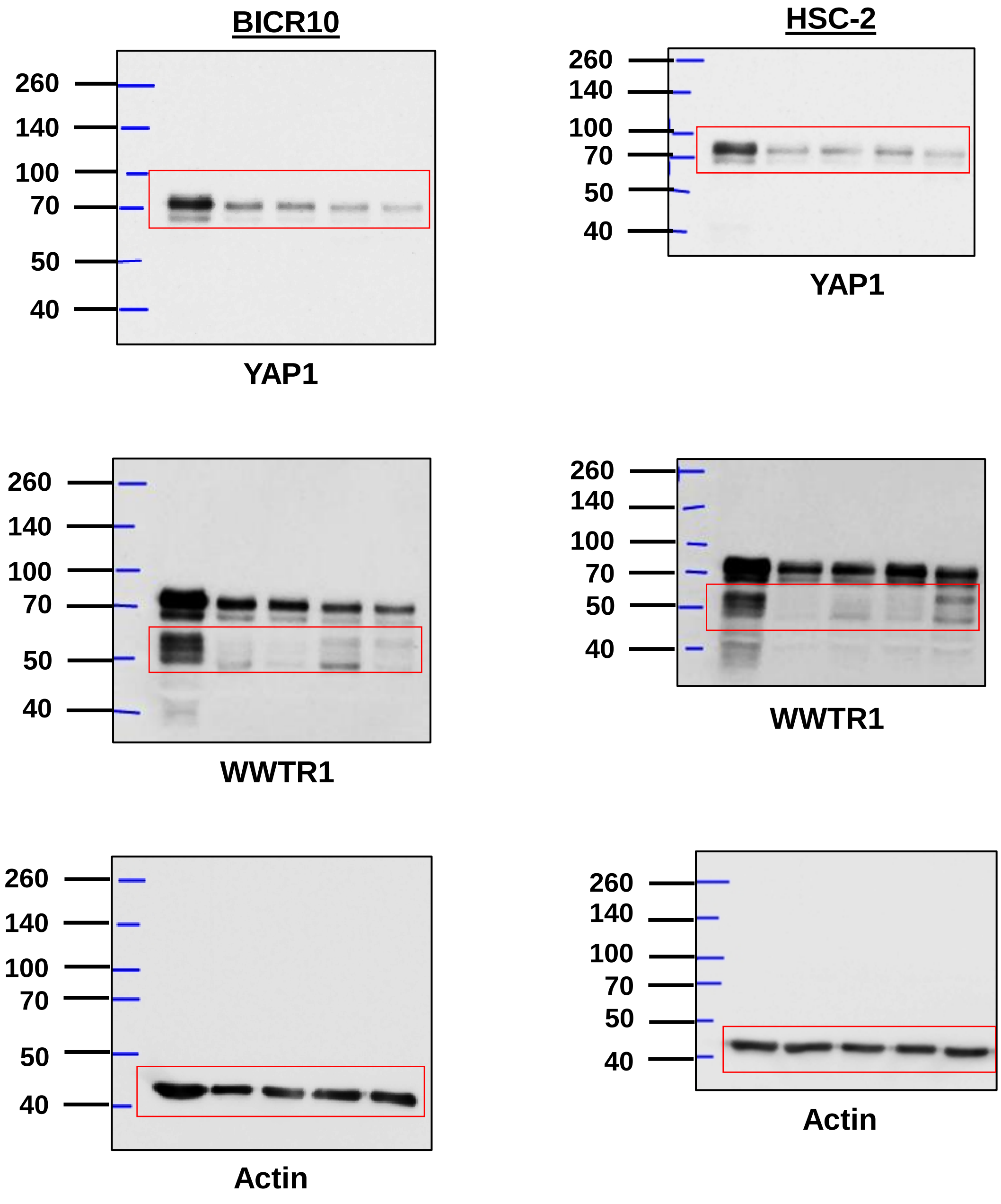

Actin was only probed on YAP1-probed blots
