# Supplementary material for: Genome-wide CRISPR screens of oral squamous cell carcinoma reveal fitness genes in the Hippo pathway
Source: eLife. 2020 Sep 29;9:e57761. doi: 10.7554/eLife.57761 (PMC7591259; doi:10.7554/eLife.57761)
Supplement: Transparent reporting form [file elife-57761-transrepform.pdf]

eLIFE
1st Floor
24 Hills Road
Cambridge CB2 1JP, UK
P 01223 855340
W elifesciences.org
T @elife

eLife's transparent reporting form

We encourage authors to provide detailed information *within their submission* to facilitate the interpretation and replication of experiments. Authors can upload supporting documentation to indicate the use of appropriate reporting guidelines for health-related research (see EQUATOR Network), life science research (see the BioSharing Information Resource), or the ARRIVE guidelines for reporting work involving animal research. Where applicable, authors should refer to any relevant reporting standards documents in this form.

If you have any questions, please consult our Journal Policies and/or contact us: editorial@elifesciences.org.

Sample-size estimation

- You should state whether an appropriate sample size was computed when the study was being designed
- You should state the statistical method of sample size computation and any required assumptions
- If no explicit power analysis was used, you should describe how you decided what sample (replicate) size (number) to use

Please outline where this information can be found within the submission (e.g., sections or figure legends), or explain why this information doesn't apply to your submission:

CRISPR screens were performed in triplicates and the minimum number of cells kept to maintain adequate representation of the sgRNA library (minimum 100x library coverage). More details can be found in the "Genome-wide CRISPR-Cas9 knockout screening" section of the Materials & Methods. Standard practice in the field was referred and statistical significance of depleted sgRNA/genes were identified using MAGeCK, a widely used algorithm in this field. A number of 21 OSCC cell lines were screened, mainly limited to the number of OSCC cell lines available in our institute and cost. Nonetheless, analysis of their genomic landscape show adequate representation of the tumor heterogeneity of OSCC.

Replicates

- You should report how often each experiment was performed
- You should include a definition of biological versus technical replication
- The data obtained should be provided and sufficient information should be provided to indicate the number of independent biological and/or technical replicates
- If you encountered any outliers, you should describe how these were handled
- Criteria for exclusion/inclusion of data should be clearly stated
- High-throughput sequence data should be uploaded before submission, with a private link for reviewers provided (these are available from both GEO and ArrayExpress)

Please outline where this information can be found within the submission (e.g., sections or figure legends), or explain why this information doesn't apply to your submission:

Number of biological or technical replicates in each experiment is described in respective figure legends, or detailed in the corresponding section under materials & methods.
Biological replicates were considered to be independent experiments performed using biological samples (cells) independent of one another, at different time. While technical replicates refer to experiments performed using cells derived from same set of biological samples but repeated times, over the same time period.
Data from the CRISPR screen, WES and RNA-seq, as well as source data for figures and their supplements are available on figshare: https://doi.org/10.6084/m9.figshare.11919753

Statistical reporting

- Statistical analysis methods should be described and justified
- Raw data should be presented in figures whenever informative to do so (typically when N per group is less than 10)
- For each experiment, you should identify the statistical tests used, exact values of N, definitions of center, methods of multiple test correction, and dispersion and precision measures (e.g., mean, median, SD, SEM, confidence intervals; and, for the major substantive results, a measure of effect size (e.g., Pearson's r, Cohen's d)
- Report exact p-values wherever possible alongside the summary statistics and 95% confidence intervals. These should be reported for all key questions and not only when the p-value is less than 0.05.

Please outline where this information can be found within the submission (e.g., sections or figure legends), or explain why this information doesn't apply to your submission:

Details of statistical analyses are outlined in corresponding figure legends, and describe in more detail in the "Statistical analysis" of the Materials & Methods section. Besides, source data file for each figure and supplementary figures are provided. Raw statistical ranking data of the CRISPR screen generated using MAGeCK software were also accessible via Figshare: **https://doi.org/10.6084/m9.figshare.11919753**

(For large datasets, or papers with a very large number of statistical tests, you may upload a single table file with tests, Ns, etc., with reference to sections in the manuscript.)

Group allocation

- Indicate how samples were allocated into experimental groups (in the case of clinical studies, please specify allocation to treatment method); if randomization was used, please also state if restricted randomization was applied
- Indicate if masking was used during group allocation, data collection and/or data analysis

Please outline where this information can be found within the submission (e.g., sections or figure legends), or explain why this information doesn't apply to your submission:

This is not applicable in this study.

Additional data files ("source data")

- We encourage you to upload relevant additional data files, such as numerical data that are represented as a graph in a figure, or as a summary table
- Where provided, these should be in the most useful format, and they can be uploaded as "Source data" files linked to a main figure or table
- Include model definition files including the full list of parameters used
- Include code used for data analysis (e.g., R, MatLab)
- Avoid stating that data files are "available upon request"

Please indicate the figures or tables for which source data files have been provided:

Source data for all relevant figures were provided:
Fig. 1a,1b,1c; Fig. 2a, 2b, 2c, 2d, 2e; Fig. 3a, 3c, 3d; Fig. 4a, 4b; Fig. 5b, 5c; Fig. 6a, 6b, 6d, 6e, 6f, 6g;

Code used for analysis is previously published and publicly accessible:
MAGeCK (version 0.5.7) - https://sourceforge.net/p/mageck/wiki/Home/
CRISPRcleanR (version 0.5) - https://github.com/francescojm/CRISPRcleanR

Public data from TCGA were accessed from cBioportal (http://cbioportal.org)
